# Aberrant expression of transglutaminase 2 in pancreas and thymus of NOD mice underscores the importance of deamidation in neoantigen generation

**DOI:** 10.3389/fendo.2022.908248

**Published:** 2022-07-26

**Authors:** Aїsha Callebaut, Ylke Bruggeman, Cloé Zamit, Fernanda Marques Câmara Sodré, Magali Irla, Chantal Mathieu, Mijke Buitinga, Lut Overbergh

**Affiliations:** ^1^ Laboratory of Clinical and Experimental Endocrinology, Department of Chronic Diseases and Metabolism, KU Leuven, Leuven, Belgium; ^2^ CNRS, INSERM, Centre d’Immunologie de Marseille-Luminy, Aix-Marseille University, Marseille, France; ^3^ Department of Microbiology, University of Sao Paulo, Sao Paulo, Brazil; ^4^ Department of Nutrition and Movement Sciences, Maastricht University, Maastricht, Netherlands; ^5^ Department of Radiology and Nuclear Medicine, Maastricht University Medical Center, Maastricht, Netherlands

**Keywords:** diabetes, neoepitopes, deamidation, post-translational modification, NOD mouse

## Abstract

Post-translational modifications can lead to a break in immune tolerance in autoimmune diseases such as type 1 diabetes (T1D). Deamidation, the conversion of glutamine to glutamic acid by transglutaminase (TGM) enzymes, is a post-translational modification of interest, with deamidated peptides being reported as autoantigens in T1D. However, little is known about how *Tgm2*, the most ubiquitously expressed *Tgm* isoform, is regulated and how tolerance against deamidated peptides is lost. Here, we report on the aberrant expression and regulation of *Tgm2* in the pancreas and thymus of NOD mice. We demonstrate that *Tgm2* expression is induced by the inflammatory cytokines IL1β and IFNγ in a synergistic manner and that murine pancreatic islets of NOD mice have higher *Tgm2* levels, while *Tgm2* levels in medullary thymic epithelial cells are reduced. We thus provide the first direct evidence to our knowledge that central tolerance establishment against deamidated peptides might be impaired due to lower *Tgm2* expression in NOD medullary thymic epithelial cells, which together with the aberrantly high levels of deamidated peptides in NOD β-cells underscores the role of deamidation in amplifying T-cell reactivity.

## Introduction

Type 1 diabetes (T1D) is a chronic autoimmune disease in which insulin-producing β-cells in the pancreatic islets of Langerhans are destroyed. Although it is well-established that autoreactive T cells play a central role in β-cell destruction, growing evidence now suggests a more complex interplay between the β-cell and the innate and adaptive immune system is underlying the pathogenesis of T1D (1). The β-cell is highly susceptible to stress; with different stress situations, such as high metabolic demand, non-specific inflammation, virus load or reactive oxygen species, provoking in particular endoplasmic reticulum (ER) and oxidative stress (2–4). Such stressors do not only play a role in β-cell dysfunction and death, but they can also induce changes in β-cell proteins, such as post-translational modifications (PTMs) (5–12), which can lead to a break in immune tolerance (6–9, 11–16). One PTM of interest in T1D is deamidation, the conversion of glutamine (Gln) to glutamic acid (Glu) by transglutaminase (TGM) enzymes in the presence of supraphysiological calcium concentrations. Among all nine distinct TGM isoenzymes identified in mammals, the most widely distributed and studied isoenzyme, tissue transglutaminase (TGM2), is expressed by almost all cell types in the body. Although a small fraction of TGM2 is localized extracellularly and in the cell membrane, the main localization of TGM2 is the cytosol (17). The murine CD4^+^ diabetogenic BDC2.5 T-cell clone was shown to have an increased interferon γ (IFNγ) response and TGM2 activity compared to exposure to control islets when exposed to murine islets in which ER stress was induced chemically by thapsigargin (18). Furthermore, TGM2 is activated during ER stress and translocates to the ER and secretory granules upon activation, a prerequisite for the observed increase in deamidation of secretory granule proteins during ER stress (18–25). Importantly, direct evidence for a role of ER stress-induced TGM2 in increasing immunogenicity of β-cell proteins has been shown in murine insulinomas (18). We have previously shown that deamidation by TGM2 occurs in β-cells (26), and data from *in vitro* systems, animal models, and human T1D point to a role for these modified proteins as neoepitopes in T1D (5–8). Deamidated GAD65, IA-2 and proinsulin C-peptide were shown to be autoantigens in T1D, with higher frequencies of memory T cells against deamidated GAD65 and deamidated IA-2 in patients compared to healthy controls and the immune response against deamidated C-peptide dominated by interleukin 10 (IL10) in controls versus IFNγ in T1D subjects (5, 8, 27). More recently, deamidated insulin C-peptide was found to be part of the immunopeptidome of NOD mice and, although CD4^+^ T cells exclusively recognizing the deamidated peptide were not present, they did enhance the immunogenicity of the native insulin C-peptide (12). How tolerance against modified peptides is lost in T1D is not fully understood. It has been suggested that PTMs not present at the time of negative selection in the developing thymus may allow T cells reactive against the modified peptides to escape negative selection, generating an autoimmune repertoire that will respond to neoantigens when present in the periphery (4). To our knowledge, the expression and activity of TGM2 has not been investigated yet in the thymus. In this study, we aimed to evaluate expression and activity of TGM2 in β-cells and medullary thymic epithelial cells (mTECs) in NOD mice to evaluate whether there is aberrant expression. This could provide a better insight in the underlying mechanisms leading to the observed increased immunogenicity of deamidated peptides in T1D. We report that NOD mice have higher levels of deamidation in the pancreatic islets of Langerhans than C57BL/6 mice and that *Tgm2* transcription is induced by cytokines. We provide proof that *Tgm2* is expressed both in the infiltrated immune cells and in pancreatic β-cells in NOD islets. Finally, we show that NOD mice have lower *Tgm2* expression and protein levels in mTECs. These results provide novel evidence that deamidation plays a role in the generation of neoepitopes in murine T1D, through a crosstalk of the immune system and β-cells.

## Materials and methods

### Cell culture and treatment

Rat INS-1E cells, a gift from Prof. Wollheim (CMU, Geneva, Switzerland), were cultured in RPMI 1640 with Glutamax (Invitrogen), supplemented with 10 mmol/l HEPES, 10% (v/v) heat-inactivated fetal calf serum (FCS), 100 U/mL penicillin, 100 μg/mL streptomycin, 1 mmol/L sodium pyruvate, and 50 μmol/L β-mercaptoethanol. Absence of Mycoplasma contamination was confirmed by PCR analysis using Venor^®^GeM Mycoplasma PCR Detection Kit (Minerva Biolabs). After plating, cells were incubated for at least 72 hours at 37°C before going in experiment. INS-1E cells were exposed to recombinant human IL1β (10 U/mL; R&D Systems), recombinant rat IFNγ (500 U/mL; R&D Systems), actinomycin D (1μg/mL; Sigma) and cycloheximide (1 μg/mL; Sigma).

### *In vitro* TGM activity assay

The activity of the TGM2 enzyme was determined with the tissue transglutaminase microassay kit (Zedira), according to the manufacturer’s instructions.

### Mice

C57BL/6 mice were obtained from the KU Leuven animal facility, where they are bred in-house. C57BL/6 mice used are genetically identical as the colony at CRL/JAX. Non-obese diabetic (NOD) mice have been inbred in our animal facility since 1989 and are housed under semi barrier conditions. For all experiments, only female mice were used. All animal manipulations were in compliance with the principles of laboratory care and approved by the Institutional Animal Ethics Committee of KU Leuven.

### Murine islet isolation and culture

Pancreatic islets of Langerhans were isolated from 6- and 10-week-old C57BL/6 and NOD mice and cultured as previously described (28). Briefly, pancreatic glands were digested with collagenase in cold Hanks’ balanced salt solution during vigorous shaking. Islets were hand-picked under a stereomicroscope after dextran gradient centrifugation for removal of exocrine tissue.

### Murine islet endocrine and immune cell sorting

Islets were dispersed in single cell suspensions by incubation in 0.0025% trypsin-EDTA (ThermoFisher) for 3 minutes at 37°C. Single dispersed islet cells were incubated for 20 minutes with Zombie Aqua viability dye (BioLegend) at room temperature in the dark and with CD45-APCeFluor780 for 20 minutes at 4°C in the dark before FACS acquisition and sorting on a BD Influx instrument.

### Murine mTEC isolation

mTECs were isolated from thymi from 6- to 8-week-old C57BL/6 and NOD mice as described in (29). Briefly, thymi from C57BL/6 and NOD mice were minced, gently agitated for release of excess thymocytes, digested with Liberase, filtered and panned on anti-CD90.2-coated plates. Cells were than stained with Fixable Viability Dye eFluor780, CD45-PECy7, MHC-II-AF488, EPCAM-BV421, BP1-APC, UEA1-biotin and streptavidin-PE. mTECs were sorted on a BD Influx instrument.

### RNA extraction and quantitative RT-PCR

Total RNA of rat INS-1E cells was extracted using the High Pure RNA Isolation Kit (Roche) and cDNA was made using oligo-d(T) and superscript II reverse transcriptase (Invitrogen). Total RNA from murine pancreatic islets and mTECs was extracted using the Single Cell RNA Purification Kit (Norgen) and cDNA synthesized with SuperScript VILO (Invitrogen). Total RNA from whole thymus was extracted using the SV Total RNA Isolation System Protocol (Promega), cDNA was synthesized similarly as cDNA from pancreatic islets and mTECs. qRT-PCR was performed using 4 pmol primers, 0.2 μL cDNA and 5 μL Fast SYBR Green Master Mix (Applied Biosystems) on a StepOnePlus RT-PCR System (Applied Biosystems). The relative fold gene expression was calculated using the delta-delta Ct method. Primers used are summarized in **Supplementary Table 1**. Normalization was done using the geometric mean of the housekeeping genes (*Actin*, *Rpl27* and *Hprt*).

### Immunofluorescence using confocal microscopy

Frozen thymic (6 week-old mice) and pancreatic (10 week-old mice) tissue sections (30 μm) from C57BL/6 and NOD mice were fixed in 2% paraformaldehyde and incubated for 10 min in a saturation buffer composed of 0.1 M Tris HCl pH 7.4, 2% Bovine serum albumin (Axday) and 0.01% Triton X-100 (Sigma). Thymic sections were stained following the previously described protocol (30). Antibodies used were Alexa Fluor 647-conjugated anti-AIRE (1:100, Clone 5H12, ThermoFisher), rabbit anti-keratin-14 (1:600, Clone Poly19053, BioLegend) and mouse anti-TGM2 (1:80, Clone SPM358, Biotechne). Rabbit anti-keratin-14 and mouse anti-TGM2 were revealed with Cy3-conjugated donkey anti-rabbit IgG (1:500, Clone Poly4064, BioLegend) and Alexa Fluor 488-conjugated goat anti-mouse IgG (1:500, ThermoFisher). Pancreatic sections were stained with APC-conjugated anti-CD3 (1:100; Clone 145-2C11, BD Pharmingen), guinea pig anti-insulin (1:80, Clone 10091379, DAKO) and mouse anti-TGM2 (1:80, Clone SPM358, Biotechne). Guinea pig anti-insulin and mouse anti-TGM2 were revealed with Alexa Fluor 488-conjugated anti-guinea pig IgG (1:500,Clone 1841755, Invitrogen) and Cy3-conjugated goat anti-mouse IgG (1:600, Clone Poly4053, BioLegend). Sections were counterstained with 1 μg/mL DAPI (BioLegend) and mounted with Mowiol (Calbiochem). Immunofluorescence confocal microscopy was performed with a Zeiss Fast-airyscan LSM880 confocal microscope. Images were analyzed with ImageJ software.

### Orbitrap LC-MS/MS

Murine islets were prepared for proteomics and run on a Q Exactive Orbitrap mass spectrometer (ThermoScientific), following our optimized protocol for accurate detection of deamidated peptides, including manual inspection of spectra, as previously described (26). Briefly, cell lysates were reduced, alkylated and quenched, followed by protein precipitation using the Wessel-Flügge method. Digestion was performed in ammonium bicarbonate pH 8 with modified trypsin (Pierce; protein/trypsin ratio 1:20 (w/w)) for 90 minutes at 37°C in the presence of 5% acetonitrile and 0.01% ProteaseMAX (Promega). Peptide mixtures were subjected to desalting with C18 ZipTip pipet tips (Millipore) and loaded on an Ultimate 3000 UPLC system (Dionex, Thermo Scientific) coupled to a Q Exactive Orbitrap mass spectrometer (Thermo Scientific). Peptides were identified by Mascot (Matrix Science) using SwissProt as a database through Proteome Discoverer 2.2, incorporating Percolator for peptide validation. Two missed cleavages were allowed, peptide tolerance was set at 5 ppm, and MS/MS tolerance at 20 mmu. Carbamidomethylation (C) was included as a fixed modification and oxidation (M) and deamidation (N/Q) were included as variable modifications.

### Statistical analysis

All data were analyzed using GraphPad Prism 8 (GraphPad, La Jolla, CA). Statistical tests used were unpaired t-test and ordinary one-way ANOVA. Unless indicated, no significant differences were observed. *p<0.05, **p<0.01, ***p<0.001 and ****p<0.0001.

## Results

### Transcriptional expression and activity of TGM2 in INS-1E β-cells is synergistically induced by IL1β and IFNγ

**Figure 1:**
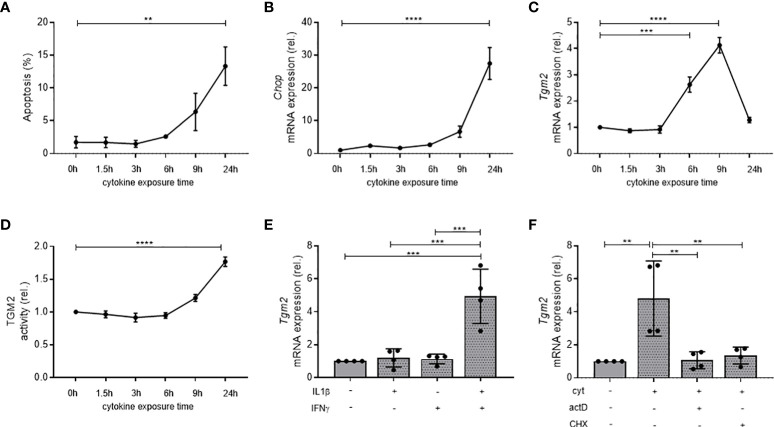
Transcription and activity of *Tgm2* in β-cells is synergistically induced, transcriptionally regulated by cytokines, and precedes cell death and ER stress. Apoptosis **(A)**, *Chop* mRNA **(B)**, *Tgm2* mRNA **(C)** and TGM2 activity **(D)** in INS-1E cells exposed to IL1β (10 U/mL) and IFNγ (500 U/mL) during different exposure times. **(E)** *Tgm2* mRNA levels in INS-1E cells exposed to IL1β and/or IFNγ for 9 hours. **(F)** *Tgm2* mRNA levels in INS-1E cells exposed to IL1β, IFNγ and actinomycin D (actD; 1μg/mL) or cycloheximide (CHX; 1μg/mL) for 9 hours. Data are shown as mean with SEM. One-way ANOVA with correction for multiple testing, **p<0.01, ***p<0.001 and ****p<0.0001.

Expression and activity of the main enzyme responsible for deamidation, TGM2, were investigated in the INS-1E β-cell line when exposed to inflammatory cytokines. Of note, out of the nine described TGM isoforms, TGM2 was the only isoform detectable in murine islets of Langerhans (data not shown), and therefore the only one further investigated in this study. For this purpose, INS-1E cells were exposed to IL1β (10 U/mL) and IFNγ (500 U/mL). In line with previous findings (31, 32), 24 hours of exposure with these cytokines resulted in increased apoptosis (13.32 vs 1.73% in control INS-1E cells, p<0.01, n=3; **Figure 1A**) and ER stress, as evidenced by increased mRNA levels of C/EBP homologous protein (*Chop*) (27.47-fold vs control INS-1E, p<0.0001, n=3; **Figure 1B**). Although apoptosis and stress were only significantly increased after 24 hours of cytokine exposure, mRNA levels of *Tgm2* were already significantly altered after 6 and 9 hours of cytokine exposure, with a 2.62- and 4.13-fold increase compared to control INS-1E cells, respectively (p<0.001 and p<0.0001, n=3; **Figure 1C**). TGM2 activity was significantly induced after 24 hours of cytokine exposure (1.77-fold vs control INS-1E, p<0.0001, n=3; **Figure 1D**). These data show that *Tgm2* is upregulated in β-cells by inflammatory cytokines and that the induction precedes cell death and ER stress. The effect of inflammatory cytokines on *Tgm2* upregulation is synergistic, as IL1β and IFNγ alone did not induce *Tgm2* upregulation after 9 hours (p < 0.001, n = 4; **Figure 1E**). Finally, inhibition of transcription (actinomycin D) and translation (cycloheximide) treatment together with the inflammatory cytokines inhibited upregulation of *Tgm2*, providing evidence that the increase in *Tgm2* is due to *de novo* transcription rather than altered stability and that this transcriptional regulation is indirectly regulated by inflammatory cytokines (p < 0.01, n = 4; **Figure 1F**). Altogether, these data show that *Tgm2* is synergistically induced in β-cells and transcriptionally regulated by inflammatory cytokines.

### NOD islets have higher *Tgm2* expression and activity, leading to an increased number of deamidated proteins in NOD compared to C57BL/6 islets

**Figure 2:**
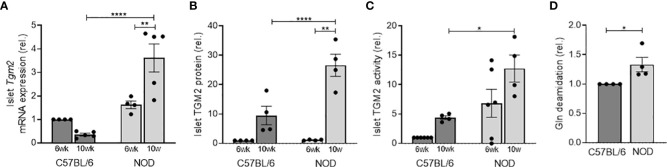
NOD mice have higher *Tgm2* RNA levels and activity in islets of Langerhans compared to C57BL/6 mice. *Tgm2* mRNA **(A)**, TGM2 protein levels **(B)** and activity **(C)** in islets of Langerhans of 6- and 10-week-old C57BL/6 and NOD mice. **(D)** Gln deamidation levels in islets of Langerhans of 10-week-old C57BL/6 and NOD mice, detected with LC-MS/MS. Data are shown as mean with SEM. One-way ANOVA with correction for multiple testing **(A–C)** and unpaired t-test **(D)**, *p<0.05, **p<0.01 and ****p<0.0001.

Given the inflammatory status in T1D in the pancreas, we then questioned whether NOD islets would have an aberrant expression of *Tgm2*. To this end, we first evaluated transcriptional expression levels of *Tgm2* in the islets of Langerhans of 6- and 10-week-old C57BL/6 and NOD mice. *Tgm2* transcription in NOD islets increased with age (p<0.01 at 6 vs 10 weeks, n = 4-5) and at 10 weeks, *Tgm2* levels were significantly higher than in C57BL/6 control islets (p<0.0001, n=5; **Figure 2A**). This was paralleled by increased TGM2 protein levels, determined by quantification of proteomics data (see below), and increased activity in 10 but not in 6-week-old NOD islets compared to C57BL/6 islets (p < 0.0001 and p < 0.05, respectively, n = 4-6; **Figures 2B**, **C**). Protein levels also significantly increased with age in NOD islets (p < 0.01, n = 4, **Figure 2B**). To further evaluate the relevance of this increased TGM2 activity, we performed LC-MS/MS analysis on protein lysates of 10-week-old C57BL/6 and NOD islets, and quantified the number of Gln to Glu deamidated peptides. For this purpose, we applied our recently optimized protocol for identifying deamidated peptides by LS-MS/MS, minimizing the level of non-enzymatically occurring deamidation by reducing trypsin digestion time, and carefully checking the spectra of the deamidated peptides to eliminate false positives (26). This revealed a 1.33-fold higher number of Gln deamidated peptides in NOD islets compared to C57BL/6 islets (304 vs 252 deamidated peptides, p < 0.05, n = 4; **Figure 2D** and **Supplementary Table 2**).

### Increased *Tgm2* levels in pancreatic islets are induced by cytokines

**Figure 3:**
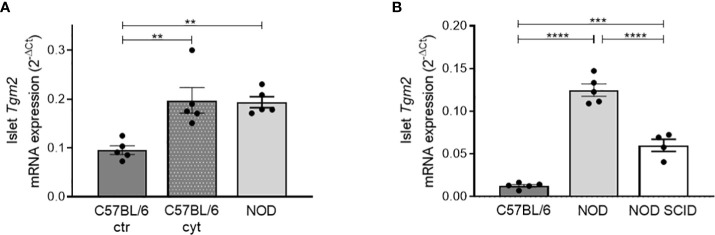
Increased *Tgm2* levels in pancreatic islets are induced by cytokines. **(A)** *Tgm2* mRNA in islets of Langerhans of 10-week-old C57BL/6 treated with or without inflammatory cytokines and NOD mice. **(B)** *Tgm2* mRNA levels in islets of Langerhans of 10-week-old C57BL/6, NOD and NOD SCID mice. Data are shown as mean with SEM. One-way ANOVA with correction for multiple testing, **p<0.01, ***p<0.001 and ****p<0.0001.

The increase in TGM2 with age in NOD mice (from 6 to 10 weeks), prompted us to investigate if this induction was induced by the cytokines produced by infiltrating leukocytes, similar to what we have seen in INS-1E cells. We therefore exposed islets of 10-week-old C57BL/6 mice, which have no leukocytes infiltrated, to IL1β and IFNγ for 24 hours and compared transcriptional *Tgm2* levels with control C57BL/6 and NOD islets, the latter having *in vivo* immune cell infiltration, leading to cytokine exposure of the β-cells. Transcriptional expression of *Tgm2* was increased two-fold in cytokine exposed C57BL/6 islets, and this to a similar level as *ex vivo* isolated NOD islets (p < 0.01, n = 5; **Figure 3A**). This finding provides evidence that inflammatory cytokines induce *Tgm2* expression in pancreatic islets. To have a better view on the extent at which the cytokines contribute to increased *Tgm2* levels in the NOD model, we compared the level of *Tgm2* expression with expression levels in islets of NOD SCID mice, defective in T and B cell development, but with functional innate immune cells. NOD SCID islets had significantly lower *Tgm2* expression compared to NOD islets, but higher than the levels measured in C57BL/6 islets (p < 0.0001 and p < 0.001 respectively, n = 5; **Figure 3B**). These results indicate that at least part of the increased *Tgm2* expression in NOD islets is mediated through the presence of infiltrating lymphocytes. Infiltrating lymphocytes may be expressing *Tgm2* themselves or could induce *Tgm2* expression in endocrine cells through cytokine secretion, or it may be a combination of both.

### Pancreatic islets express *Tgm2* both in endocrine and infiltrating immune cells

**Figure 4:**
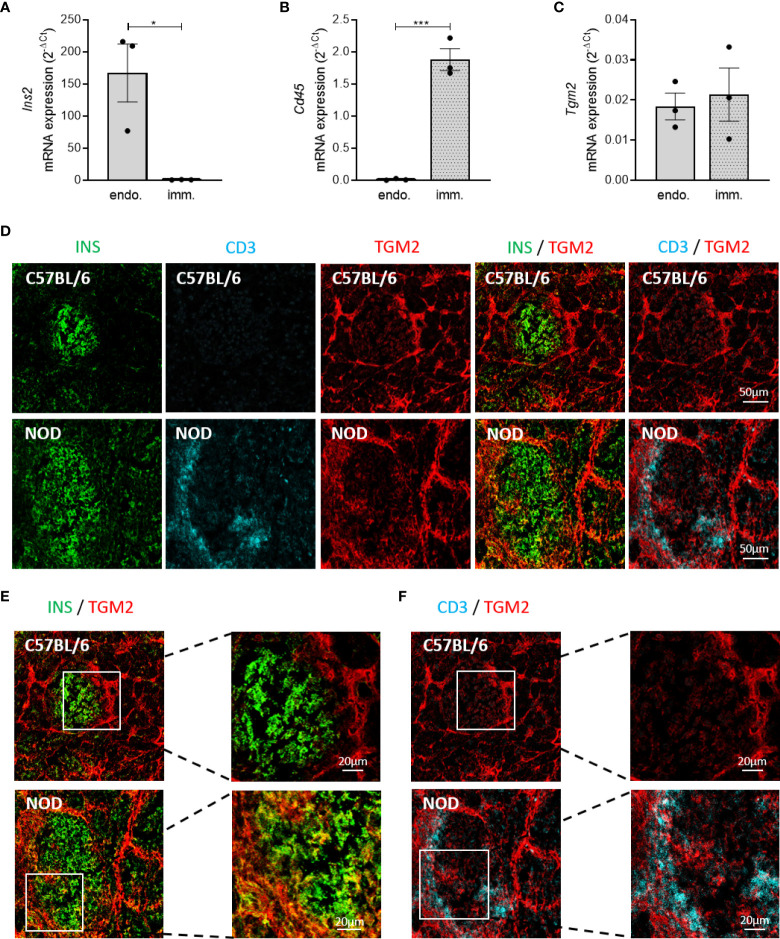
Increased *Tgm2* levels in islets of Langerhans of NOD mice are induced by cytokines. *Tgm2* is expressed by both β-cells and infiltrating immune cells. *Tgm2* RNA levels in control C57BL/6 islets and C57BL/6 islets exposed to IL1β and IFNγ for 24 hours. *Tgm2 Ins2* **(A)**, *Cd45* **(B)** and *Tgm2 Ins2* **(C)** mRNA levels in CD45^+^ (immune) and CD45^-^ (endocrine) sorted fractions of NOD islets. **(D)** TGM2 protein expression in murine islets of Langerhans. Confocal microscopy of frozen pancreas tissue section from 10-week-old C57BL/6 and NOD mice. Immune cells are visualized by CD3 staining, β-cells by insulin. **(E)** Zoom in of INS and TGM2 merged image in C57BL/6 and NOD islets. **(F)** Zoom in of CD3 and TGM2 merged image in C57BL/6 and NOD islets. Data are shown as mean with SEM. Unpaired t-test, *p < 0.05, ***p < 0.001.

Next, to elucidate whether it are the endocrine and/or the infiltrated immune cells that are responsible for the expression of *Tgm2* within NOD islets, we quantified *Tgm2* transcripts by qRT-PCR in sorted endocrine and immune cells of 10-week-old NOD islets cells. Endocrine and immune cells were separated based on CD45 negative and positive fractions using flow cytometry (gating strategy depicted in **Supplementary Figure 1A**). The purity of both fractions was confirmed by the absence of insulin 2 (*Ins2*) and *Cd45* in the immune and endocrine fractions, respectively (p < 0.001 and p < 0.05, n = 3; **Figures 4A**, **B**). Purity was further confirmed by the absence of glucagon and somatostatin in the immune fraction and the absence of the endothelium marker *Cd31* in both populations (**Supplementary Figures 1B**–**D**). In both immune and endocrine cells, *Tgm2* was clearly expressed, with no significant difference in expression level between both cell populations (n = 3; **Figure 4C**). To extend this finding to the protein level, we performed immunohistochemistry to evaluate the distribution of TGM2 protein expression in pancreas slides from 10-week-old C57BL/6 and NOD mice. Only in NOD islets, TGM2 staining was observed in some insulin-positive cells (**Figures 4D**, **E**). The fact that these cells were typically located nearby the immune cell infiltrate supports our observation that the inflammatory environment enhances TGM2 expression in β-cells. In addition, TGM2 also co-localized with infiltrating CD3 positive T cells in NOD islets (**Figure 4F**), in line with the observed transcriptional expression in the FACS sorted CD45 positive cell fraction. Of note, also a clear expression of TGM2 was observed in blood vessels surrounding the islets, both in C57BL/6 and NOD islets, as described in literature, where TGM2 is present at endothelial cell-cell and cell-matrix contact points and activation results in increased permeability (33). Taken together, the increased expression of *Tgm2* in the NOD islet is likely a consequence of *Tgm2* expression in the infiltrating immune cells as well as increased *Tgm2* expression in the pancreatic β-cells exposed to cytokines secreted by these infiltrating immune cells.

### *Tgm2* is expressed and active in the thymus of C57BL/6 and NOD mice, but reduced in mTECs of NOD mice

**Figure 5:**
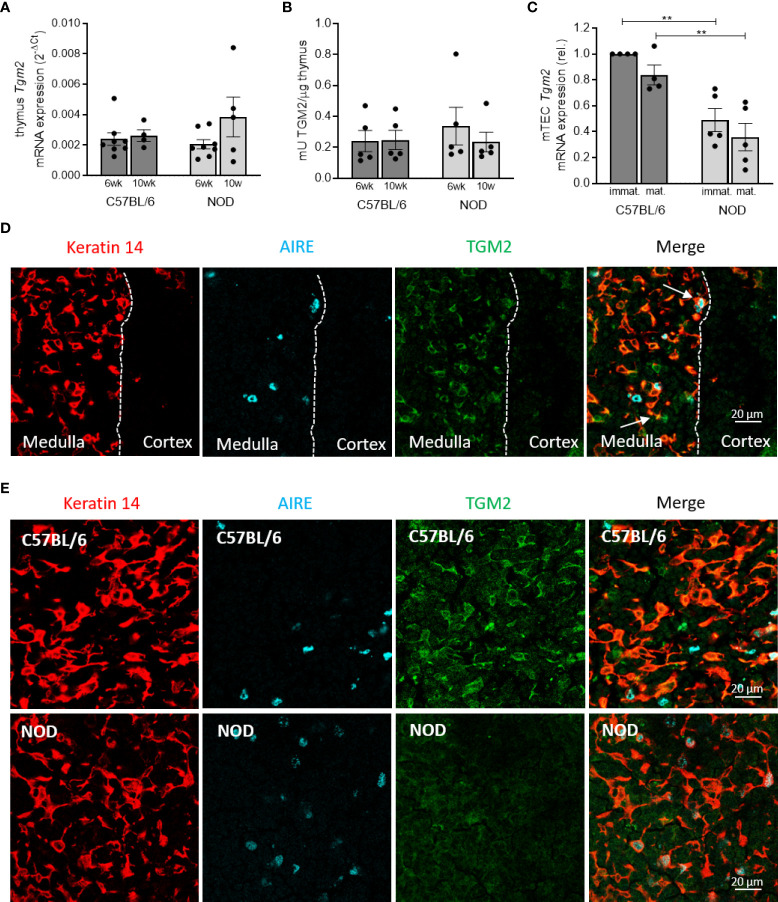
Deamidation is present in the thymus but TGM2 expression is reduced in mTECs of NOD mice. *Tgm2* mRNA levels **(A)** and TGM2 activity **(B)** in the thymus of 6- and 10-week-old C57BL/6 and NOD mice. **(C)** *Tgm2* mRNA levels in immature (MHC-II^low^) and mature (MHC-II^high^) mTEC cells of C57BL/6 and NOD mice. **(D)** TGM2 protein expression in medulla and cortex of frozen thymic tissue sections of C57BL/6 mice. mTECs are visualized by keratin 14 staining, mature mTECs by AIRE. Dashed line represents the border of medulla and cortex. TGM2 co-localization in immature (yellow) and mature (white) mTECs are indicated with arrows. **(E)** TGM2 protein expression in murine mTECs in frozen thymic tissue sections of C57BL/6 and NOD mice. mTECs are visualized by keratin 14 staining, mature mTECs by AIRE. Data are shown as mean with SEM. One-way ANOVA with correction for multiple testing, **p<0.01.

To investigate if *Tgm2* is expressed and active in the thymus and to provide further insight on the mechanism underlying the reported immunogenicity of deamidated peptides in T1D, we investigated transcriptional expression and activity levels of *Tgm2* in the thymus of 6- and 10-week-old C57BL/6 and NOD mice. Evaluation of expression of *Tgm2* in whole thymus revealed a clear transcriptional expression and activity both in the thymus of C57BL/6 and NOD mice. However, no differences in expression levels or activity were observed between C57BL/6 and NOD thymus, both at 6 and 10 weeks of age (n = 5-8; **Figures 5A**, **B**). To further define the thymic expression of TGM2 in more detail, we performed immunohistochemistry on thymus sections of C57BL/6 mice. This revealed that TGM2 was highly expressed in the medulla, and almost absent in the cortex. Within the medulla, TGM2 co-localized both with keratin 14 positive cells and with AIRE positive cells, suggesting expression both in immature MHC-II^low^ and mature MHC-II^high^ mTEC cells (**Figure 5D**). We then compared TGM2 expression between C57BL/6 and NOD thymic sections. TGM2 expression was clearly reduced in mTECs of NOD mice (**Figure 5E**). To confirm this finding quantitatively, we next asked whether *Tgm2* was differentially expressed in sorted mTECs of C57BL/6 and NOD mice (gating strategy depicted in **Supplementary Figure 2**). In mTECs, *Tgm2* expression was substantially lower in NOD mice in both immature MHC-II^low^ and mature MHC-II^high^ mTECs compared to C57BL/6 mice (p < 0.01, n = 4-5; **Figure 5C**), suggesting that the reduced *Tgm2* expression in NOD mTECs is masked in the whole thymus due to the presence of other thymic cells. These results show that the enzyme responsible for deamidation is expressed in the thymus of both C57BL/6 and NOD mice, suggesting that negative selection against deamidated peptides should be able to occur, although to a much lesser extent in NOD mice due to lower *Tgm2* expression in the mTECs.

## Discussion

We report on the aberrant expression and regulation of TGM2, one of the enzymes responsible for deamidation, in the pancreas and thymus of NOD mice. We demonstrate that TGM2 expression is induced by the inflammatory cytokines IL1β and IFNγ in a synergistic manner and that murine pancreatic islets of NOD mice have higher TGM2 levels, while TGM2 levels in thymic mTECs are reduced. We thus provide the first direct evidence to our knowledge that central tolerance establishment against deamidated peptides might be impaired due to lower *Tgm2* expression in NOD mTECs, which together with the aberrantly high levels of deamidated peptides in NOD β-cells underscores the role of deamidation in amplifying T-cell reactivity.

TGM enzymes are relatively short-lived proteins, with a half-life of about 11 hours, and are described to be highly transcriptionally regulated (17). We here demonstrate that *Tgm2* expression is induced by cytokines at the transcriptional level, in an indirect way, as cycloheximide is able to inhibit the transcriptional induction. The human *TGM2* promotor contains several transcriptional regulatory elements and transcription factor-binding sites, with CAAT, TATA, Sp1 and NF-κB binding sites among others (34). In the T84 human enterocytic cell line, IFNγ was shown to activate extracellular TGM2 and the activation required phosphatidylinositol-3-kinase (PI3K) (35). Our data suggest that IFNγ alone is not sufficient for intracellular TGM2 activation, at least not in the dose used, as co-stimulation with IL1β is necessary for its induction. Binding of NF-κB to the *TGM2* promotor can strongly stimulate enzyme induction, as shown in rat hepatocytes (36). Since both IL1β and IFNγ activate the NF-κB pathway in a different way, the observed *TGM2* induction by inflammatory cytokines might be transcriptionally regulated through NF-κB. Alternatively, the *TGM2* induction might be regulated through *IRF1*, a direct response gene of IFNγ, since an IRF1 binding site is present in the *TGM2* promotor (34). In the rat insulin producing RIN cell line, IFNγ induces *IRF1* mRNA transcription after 30 minutes and an IRF1 protein content increase after 2 hours, and this effect is further potentiated by IL1β (37).

It has previously been shown that TGM2 is activated in β-cells by ER stress, associated with increased calcium levels (18). Thapsigargin, a very strong inducer of acute ER stress with high calcium fluxes, significantly increased BDC2.5 effector responses and TGM2 activity was crucial for the increased immunogenicity, since silencing of *Tgm2* expression by shRNA resulted in lower BDC2.5 responses compared to control cells incubated with thapsigargin (18). Interestingly, BDC2.5 effector responses were also increased by physiologically stressed primary islets, although to a much lower extent (18). This suggests that exposure of β-cells to additional triggers of ER stress besides physiological ER stress already present in the β-cell may further increase TGM2 activity, deamidation of β-cell proteins and presentation of deamidated peptides to immunogenic T cells (18). We previously showed that murine MIN6 β-cells, when exposed to inflammatory cytokines, a more physiological ER stressor, had increased *Tgm2* expression, activity and levels of deamidation (26). We show here for the first time that *Tgm2* expression and activity is transcriptionally regulated in β-cells when induced by inflammatory stress, and that diabetes-prone NOD mice have increased expression, protein levels and activity of TGM2 as well as increased levels of deamidation in the pancreatic islets compared to C57BL/6 mice. Interestingly, this increase was only present at 10 weeks of age, suggesting that targeting native antigens leads to increased immune infiltration, driving cytokine production and increased ER stress, which then results in an increased TGM2 activity and deamidation of β-cell proteins, thus suggesting that deamidation is involved in the amplification rather than the initiation stage of T1D.

We report that *Tgm2* is expressed in both the endocrine cells and the immune cells infiltrating the NOD islet. The observation that cytokine exposure of C57BL/6 islets, which do not have infiltrating immune cells producing inflammatory cytokines, results in increased *Tgm2* expression to a similar extent as *Tgm2* expression in NOD islets, supports our notion that it is the inflammatory environment that leads to increased *Tgm2* expression. Furthermore, NOD SCID islets, lacking adaptive immunity, had intermediate levels of *Tgm2* in their islets. These data demonstrate that inflammatory cytokines, produced by infiltrating lymphocytes in the islets of Langerhans of NOD mice and thus an *in vivo* relevant stressor, also resulted in increased TGM2 activity, which can result in increased deamidation of β-cell proteins. TGM2 translocates to the ER and secretory granules upon activation, a prerequisite for the observed increase in deamidation of secretory granule proteins during ER stress (19–25). Although the main localization of TGM2 is the cytosol, a small fraction of TGM2 is localized in the cell membrane and extracellularly (17), suggesting that deamidation can also occur extracellularly. In addition, our data show that also immune infiltrating cells in the NOD islets are expressing TGM2. This is in line with the reported expression and cytokine-mediated induction of TGM2 in dendritic cells (38). In addition, other immune cells, such as neutrophils have been reported to express TGM2 (39). Moreover, TGM2 expression is induced in neutrophils that infiltrated the site of inflammation, compared to neutrophils in the blood and bone marrow (39). Our findings that increased TGM2 expression in NOD islets is only measurable at 10 weeks of age, and not at younger age (6 weeks), is in line with the hypothesis of inflammation-driven increase of TGM2, and fits with our *in vitro* findings that TGM2 is transcriptionally induced by cytokines. This regulation is different from what we have reported earlier for PADI2, the enzyme responsible for citrullination, in NOD islets. Indeed, we showed that *Padi2* mRNA levels and PADI2 activity were already elevated in islets of NOD mice as early as at 3 weeks of age (10). Although these findings suggest that both citrullination and deamidation are happening in islets of NOD mice, the mechanisms leading to this aberrantly high expression are different, with an intrinsic difference in citrullination and an inflammation-driven difference in deamidation.

The expression pattern of TGM2 in inflamed β-cells and NOD islets as described here is fully in line with the described role of deamidated peptides as neoepitopes in T1D (5, 8, 27). Interestingly, one of the deamidations we detected in murine islets of Langerhans was 78 kDa glucose-regulated protein (GRP78) (**Supplementary Table 2**); a protein we have shown to be an autoantigen in murine and human T1D when citrullinated (10, 11). GRP78 Q137 and Q510 were only detected in the deamidated form in NOD and not in C57BL/6 islets. GRP78 Q450, although present in both C57BL6 and NOD islets, has also been detected by us in stressed human islets (Callebaut et al., unpublished data). These findings, together with our earlier report that GRP78 translocates to the cell surface and is secreted upon inflammatory cytokine exposure (40), makes GRP78 an interesting novel autoantigen candidate in its deamidated form. Deamidation of Q62 of proinsulin C-peptide was also detected, although only in C57BL/6 islets, in line with published findings in human islets (38).

Despite the evidence that deamidated β-cell proteins are neoepitopes in T1D, little is known about the mechanism by which immune tolerance is breached towards these proteins. To this end, we here investigated the expression of TGM2 in the thymic epithelial cells of NOD mice and control C57BL/6 mice. Our observation that TGM2 was clearly expressed in the thymus of control C57BL/6 mice, almost exclusively in mTEC cells while expression was absent in the cortex, goes against the current hypothesis that PTMs are not happening in the thymus (4). Peptidylarginine deiminase (PADI) enzymes, responsible for citrullination, were recently shown by us to be expressed in both human and murine medullary epithelial cells (mTECs), which play an essential role in the establishment of central tolerance through promiscuous gene expression (PGE), also contradicting the current hypothesis (29). However, we reported a diminished expression of PADI2 in mature mTECs of NOD mice, compared to control C57BL/6 mice (29). We now showed that the NOD mTECs have lower levels of TGM2 expression, which could allow an increased escape of T cells reactive against deamidated peptides, a second PTM involved in the pathogenesis of T1D. In contrast to our findings on an AIRE-independent mTEC expression of *Tgm2*, *Padi* expression in mTECs was shown to be under the control of AIRE and the effect of AIRE on PGE may thus extend to citrullinated proteins (29). Many tissue-restricted antigens expressed in mTECs are however described as being AIRE-independently regulated, implying that additional factors can also regulate PGE and that deamidation could be under the control of one of those other factors (41). Furthermore, we also showed that the NOD thymus harbored fewer MHC-II^high^ mature mTECs, contributing to defective central tolerance in the NOD mice in general (29). A similar mechanism as seen in NOD mice might occur in human T1D, where genetically susceptible individuals might have defective tolerance mechanisms against deamidation and other PTMs in the thymus. In favor of this hypothesis, we have recently shown that *TGM2* is expressed in human MHC-II^low^ and MHC-II^high^ mTECs (29). It would therefore be relevant to investigate if there are differences in *TGM2* expression in mTECs and pancreatic islets of Langerhans between healthy individuals and subjects with T1D.

In conclusion, the present findings increase our understanding of the underlying mechanisms leading to a loss of tolerance against PTM epitopes, in particular deamidated epitopes, in an autoimmune T1D setting. As such, increased deamidation through inflammation in NOD islets of Langerhans in combination with defective negative tolerance mechanisms against deamidated peptides may drive autoimmunity against deamidated β-cell peptides.

## Data availability statement

The datasets presented in this study can be found in online repositories. The names of the repository/repositories and accession number(s) can be found below:

PRIDE database, PXD034102

## Ethics statement

The animal study was reviewed and approved by Animal Ethics Committee of KU Leuven KU Leuven, Belgium.

## Author contributions

AC, YB, CZ, FM, CS, MI and MB contributed to the design, conduct, analysis and interpretation of the data. The manuscript was written through contributions of all authors. All authors have given approval to the final version of the manuscript. CM and LO are the guarantors of this work and, as such, had full access to all the data in the study and take responsibility for the integrity of the data and the accuracy of the data analysis. All authors contributed to the article and approved the submitted version.

## Funding

This work was supported by IMI2-JU under grant agreement No 115797 (INNODIA) and No 948268 (INNODIA HARVEST). This joint undertaking receives support from the Union’s Horizon 2020 research and innovation program and EFPIA, JDRF and The Leona M. and Harry B. Helmsley Charitable Trust; the KU Leuven (C16/18/006) and the Flemish Research Foundation (a predoctoral fellowship for A.C. (1189518N) and Y.B. (1179921N) and a postdoctoral fellowship for MB (3-PDF-2018-593-A-N). This work was supported by the French National Research Agency through the “Investments for the Future” program (France-BioImaging, ANR-10-INBS-04).

## Acknowledgments

The authors thank Martine Gilis, Jos Laureys, Marijke Viaene, Marc Packbier and Eline Desager (KULeuven, Leuven, Belgium) for expert assistance and the SyBioMa Mass Spectrometry Facility of KULeuven. The authors thank the KULeuven Flow Cytometry Core Facility for assistance with flow cytometry. We thank the imaging core facility (ImagImm) of the Centre d’Immunologie de Marseille-Luminy (CIML).

## Conflict of interest

The authors declare that the research was conducted in the absence of any commercial or financial relationships that could be construed as a potential conflict of interest.

## Publisher’s note

All claims expressed in this article are solely those of the authors and do not necessarily represent those of their affiliated organizations, or those of the publisher, the editors and the reviewers. Any product that may be evaluated in this article, or claim that may be made by its manufacturer, is not guaranteed or endorsed by the publisher.
