# Supplementary figures and images for: Aberrant expression of transglutaminase 2 in pancreas and thymus of NOD mice underscores the importance of deamidation in neoantigen generation
Source: Front Endocrinol (Lausanne). 2022 Jul 26;13:908248. doi: 10.3389/fendo.2022.908248 (PMC9367685; doi:10.3389/fendo.2022.908248)

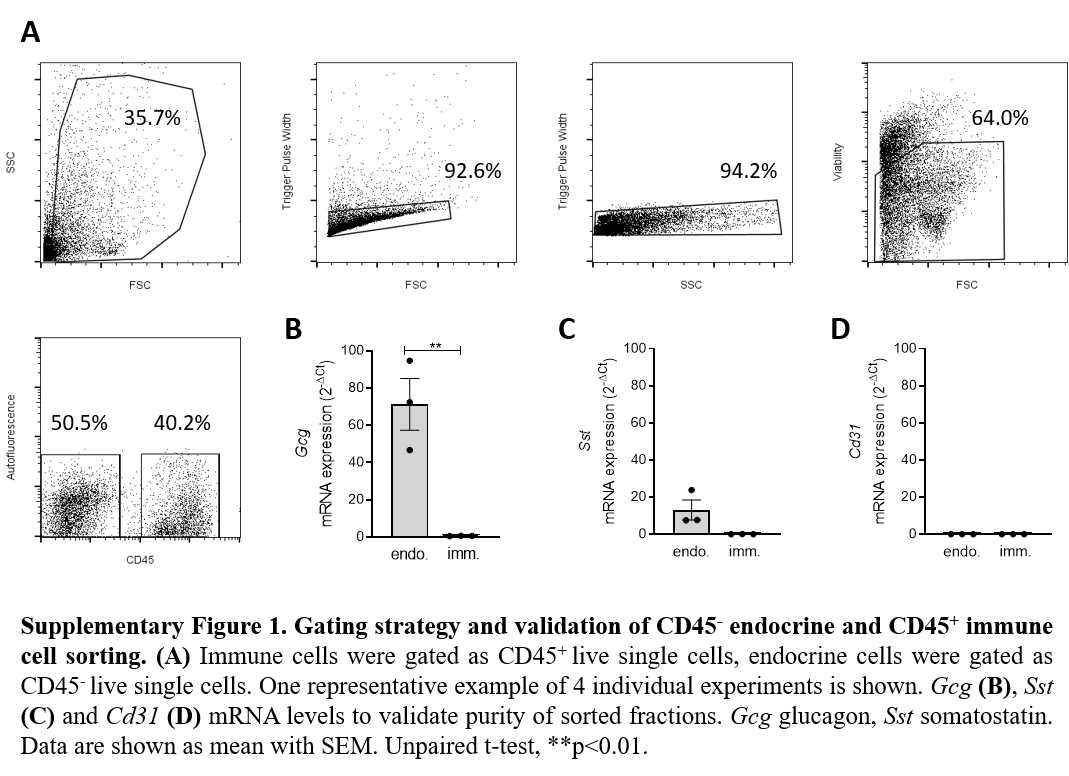

Supplement: Supplementary file 1 [file Image_1.tif]

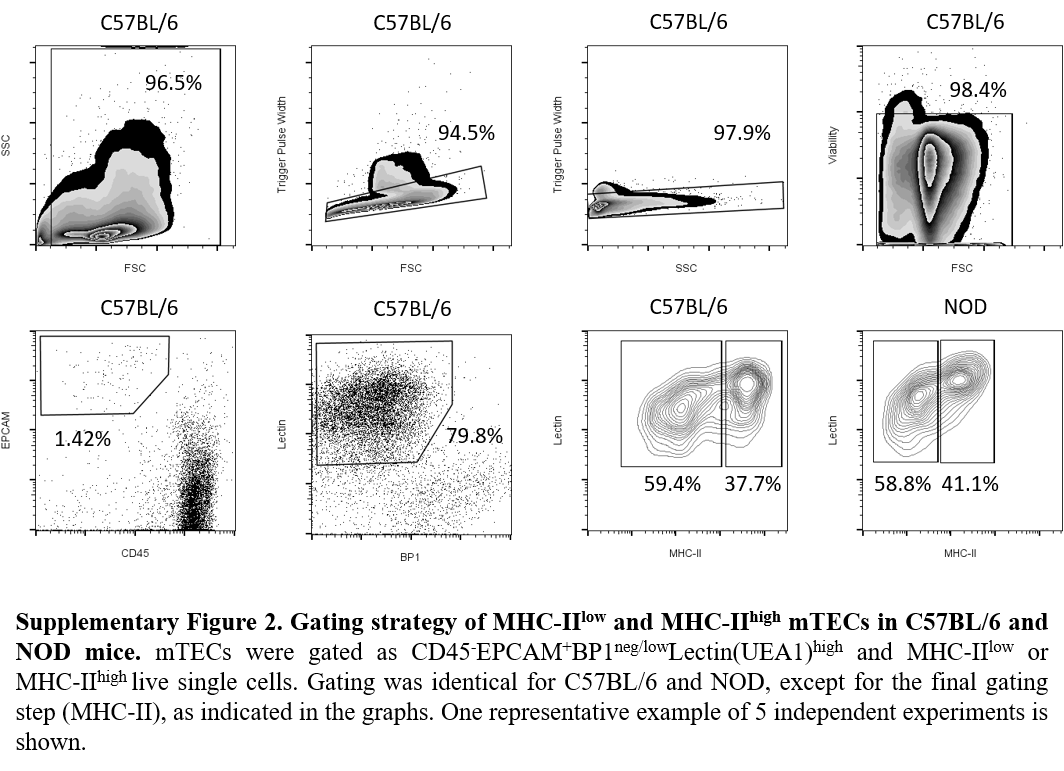

Supplement: Supplementary file 2 [file Image_2.tif]
